# Rapid progression to end-stage renal disease in a child with a sporadic ACTN4 mutation 

**DOI:** 10.5414/CNCS108616

**Published:** 2015-09-23

**Authors:** Aadil K. Kakajiwala, Kevin E. Meyers, Tricia Bhatti, Bernard S. Kaplan

**Affiliations:** 1 Division of Nephrology and; 2 Division of Pathology, The Children’s Hospital of Philadelphia, Philadelphia, PA, USA

**Keywords:** ACTN4 gene mutation, focal segmental glomerulosclerosis, end-stage renal disease

## Abstract

Mutations of ACTN4 cause an autosomal dominant form of focal segmental glomerulosclerosis (FSGS). Presentation usually occurs in the teenage years or later with symptoms of mild proteinuria and slowly progressive renal dysfunction leading to end-stage renal disease (ESRD). We report a 5-year-old female patient who was diagnosed with nephrotic syndrome and did not respond to 6 weeks of oral glucocorticoid therapy. Renal biopsy showed a collapsing variant of FSGS and genetic studies revealed a heterozygous disease-causing mutation in the ACTN4 gene (c.784C>T, p.Ser262Phe). No mutations were found in the NPHS2, TRPC6, and INF2 genes, nor did her parents have any mutations for FSGS. She developed ESRD 6 months after presentation. Although a disease-causing ACTN4 mutation was identified, the contribution of additional polymorphisms in other genes is not known. Such additional polymorphisms may represent yet unidentified epigenetic factors that contributed to the aggressive nature of this child’s disease progression. A literature review has revealed only two similar case reports.

## Introduction 

Mutations in the gene encoding alpha-actinin-4 (ACTN4) are the leading causes of autosomal dominant form of focal segmental glomerulosclerosis (FSGS) accounting for ~ 2 – 4% of cases of familial FSGS [1]. Multiple ACTN4 sequence variants have been identified with several predicted to alter the encoded amino acid sequence of the protein [2, 3]. 

Patients with mutations in ACTN4 typically present in the teenage years or later and have slow progression of renal dysfunction. Some may progress to end-stage renal disease (ESRD), usually in the fifth decade of life [2, 4]. 

This report illustrates a case of a collapsing form of FSGS secondary to sporadic ACTN4 mutation that rapidly progressed to ESRD. A literature review revealed one case report of two siblings with p.Ser262Phe mutation in the ACTN4 gene and p.Thr5Met substitution in NPHS1 gene (of unknown significance). Both patients presented with NS in early childhood, one of whom rapidly progressed to ESRD. Renal biopsies showed collapsing glomerulopathy in one patient and FSGS not otherwise specified in the other. Their father, who was clinically silent, possessed a germline mosaicism of the mutation [5]. Wein et al. [1, 2] described the case of a 5-year-old male with an ACTN4 mutation (p.Trp59Arg substitution) who developed ESRD within 3 years of diagnosis. This patient however, experienced recurrence of his proteinuria 2 years after transplantation that had initially responded to plasmapheresis. A biopsy of the transplanted kidney showed non-specific interstitial fibrosis and tubular atrophy, but the possibility of recurrent FSGS could not be excluded. 

## Case presentation 

A 5-year-old Caucasian female presented with abdominal pain and emesis. She had experienced facial puffiness for 2 weeks prior to onset of other symptoms. On examination, she was found to be febrile with tachycardia, anasarca, and described diffuse abdominal tenderness. Laboratory studies showed an elevated white cell count of 38.5 × 10^3^/µL with 87% segmented neutrophils and 5% bands, normal serum electrolytes, a serum creatinine concentration of 0.68 mg/dL, and a serum albumin concentration of < 1 g/dL. The urinalysis was positive for 4+ proteinuria and moderate blood, but no red cell casts were seen. A CT scan revealed bowel wall thickening and free fluid in the abdomen. At the time of admission, a blood hemoglobin concentration 9 g/dL was recorded and the blood cultures were positive for *Streptococcus pneumoniae*. The patient was diagnosed with bacteremia secondary to peritonitis from new onset nephrotic syndrome and was started on glucocorticoid therapy along with a course of intravenous cefotaxime followed by oral levofloxacin. 

**Figure 1.**
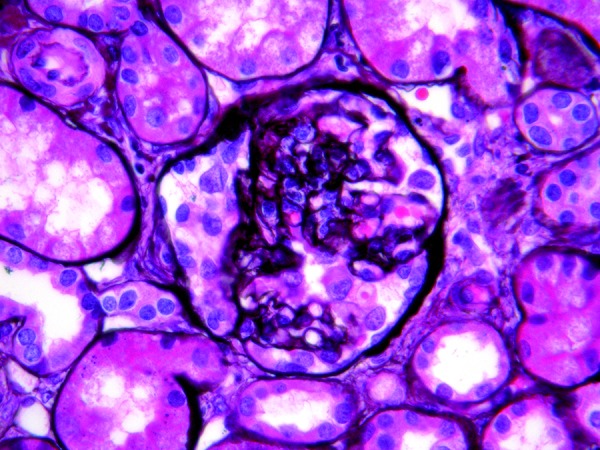
Jones silver stain ×630 original magnification. Histologic sections of the majority of glomeruli showed variable degrees of collapse of the glomerular tufts associated with visceral epithelial cell (podocyte) proliferation. Tubular atrophy, patchy interstitial inflammation, and fibrosis were also present.

The patient showed no response after six weeks of daily glucocorticoid therapy. A renal biopsy revealed variable degrees of wrinkling and collapse of the basement membranes within thirteen of the 32 glomeruli as well as occlusion of the capillary loops. Two of these glomeruli were globally sclerosed. Proliferation of visceral epithelial cells was apparent (Figure 1). One core showed pronounced interstitial fibrosis with tubular atrophy while other areas of the interstitial showed patchy chronic inflammation and interstitial fibrosis. Immunofluorescent microscopy was weakly positive for granular deposits of IgM, C3, and C1q in the mesangium. Ultrastructural studies showed diffuse foot process effacement, multiple foci of membrane thickening, and consolidation of capillary tufts. 

DNA sequence analysis was performed by PCR amplification of highly purified genomic DNA, followed by automated bi-directional DNA sequencing of the coding regions of the NPHS2, ACTN4, TRPC6, and INF2 genes. Of the INF2 gene, which has 22 exons, exon 1 is non-coding and thus was not included in the analysis. Additionally, only the 3’ end of exon 8 was analyzed. In exons where one of the strands was not informative for confirmation, a uni-directional sequencing approach with alternative dye chemistry was used for confirmation. At least 20 bases of intronic DNA surrounding each exon were also sequenced. Genetic studies showed a heterozygous disease-causing mutation in the ACTN4 gene (c.784C>T, p.Ser262Phe) (Table 1A) with no mutations found in the NPHS2, TRPC6 and INF2 genes. Similar DNA analysis on the patient’s parents revealed no mutations in these four genes. The patient developed severe hypertension and posterior reversible encephalopathy syndrome (PRES) while taking tacrolimus and corticosteroids, which were then discontinued. She did not respond to a course of four doses of Rituximab, each 375 mg/m^2^. Blood pressure was controlled with a regimen of amlodipine, furosemide, losartan, and metolazone. Six months after presentation, the patient progressed to ESRD and started on peritoneal dialysis for hyperkalemia, acidosis, and fluid overload. After being managed on peritoneal dialysis for 5 months, she underwent a living unrelated donor transplant and has not experienced a recurrence of disease. 

## Discussion 

Podocytes are terminally differentiated and highly specialized epithelial cells that cover the outer layer of the glomerular basement membrane (GBM). The podocyte and mutations/polymorphisms in podocyte-specific genes are central to the pathogenesis of FSGS [6]. Podocytes have an actin-based contractile apparatus that links their foot processes to each other. The actin cytoskeleton is an essential structural and functional element that helps controls cell shape, motility, and adhesion. The assembly and maintenance of the actin cytoskeleton is mediated by a variety of actin-associated proteins. ACTN4, an actin-binding and cross linking protein that localizes to the renal glomerulus, encodes predominantly at the foot processes [1, 4, 7]. It has important roles both in cross-linking of actin filaments into contractile bundles and in helping to form the anchoring complex for the ends of actin stress fibers. Mutations in α-actinin-4 increase its affinity for actin, alter the mechanical properties of the podocyte, and lead to familial form of FSGS. 

FSGS is defined by involvement of some portion of the tuft (segmental) of some glomeruli (focal) in sclerosis accounting for ~ 20% of all the causes of nephrotic syndrome in children [8]. FSGS is primarily a disease of the podocyte and should be thought of as a podocytopathy [9]. There are primary (idiopathic) and secondary forms of FSGS, with primary disease constituting ~ 80% of all cases. An increasing list of autosomally dominant inherited forms of FSGS include mutations in INF2 (encoding inverted formin 2), TRPC6 (encoding a transient receptor potential ion channel) and ACTN4 genes. Autosomal recessive inheritance is usually the result of a mutation in NPHS2 (encoding podocin) and is quantitatively more common. Other mutations associated with development of FSGS involve mutations in the WT1, LAMB2, ARGHGAP24, CD2AP, phospholipase C epsilon 1, myosin 1E genes [8, 10]. The high rate of FSGS in patients of recent African ancestry is largely attributable to two variants in the APOL1 gene [3, 11]. Genetic testing can help determine risk of FSGS recurrence after renal transplant and allow for risk assessment in living candidates living related to kidney donors. To date, there has only been one reported case of possible recurrence of FSGS in a patient with W59R-ACTN4 mutation [1, 2, 3]. That patient presented with proteinuria and a normal serum creatinine at 5 years of age and progressed to ESRD 3 years later. In addition, the patient had non-syndromic bilateral sensorineural deafness. He received a kidney transplant from his father, but developed recurrence of proteinuria that responded to plasmapheresis. His kidney function declined over 2 years and a transplant biopsy showed interstitial fibrosis and tubular atrophy, but the possibility of FSGS could not be excluded. 

In 2000, Kaplan et al. [1] discovered that an in-vitro mutant α-actinin-4 bound to filamentous actin more strongly than wild-type α-actinin-4, affecting regulation of the podocyte actin cytoskeleton. A study by Yao et al. [12] in 2004 suggested that aggregation of large mutant α-actinin protein and the degradation of mutant protein over decades ultimately proves toxic. Hence, individuals with ACTN4-associated FSGS have focal podocyte abnormalities and slowly progressive teenage or adult-onset disease, leading to significant renal failure in adulthood. Henderson et al. [4], who studied the histopathology of renal biopsies of patients with ACTN4 mutation, showed morphologically heterogeneous features, typical of FSGS, including global sclerosis, segmental sclerosis, glomerular hypertrophy and presence of mesangial expansion. The ultrastructural characteristics, however, were distinctive. Most notable was the presence of irregularly aggregated electron-dense aggregates in the podocyte cytoplasm, usually seen in podocyte major processes or cell bodies, and was almost always associated with the cell membrane. Collapsing glomerulopathy is characterized by segmental or global wrinkling of the glomerular basement membranes associated with podocyte proliferation. Patients with collapsing glomerulopathy (Table 1B) are more likely to have massive proteinuria, have a higher likelihood of baseline renal insufficiency, progress to ESRD, and are particularly poor responders to standard therapies [13, 14]. 

We did not perform a functional study of the p.Ser262Phe substitution in ACTN4 as this same amino acid substitution has been described in prior studies. Both parents did not have mutation of ACTN4 gene and were asymptomatic, suggesting that this variant was truly disease causing. The unique features of this case are the presentation in early childhood, the presence of a collapsing form of FSGS, and the rapid progression to ESRD. ACTN4 mutations usually manifest in adult life, though interestingly, not all persons carrying disease-associated ACTN4 mutations develop clinical disease. It has been suggested that a second hit, representing either a second mutation in another gene or a physiological stressor may induce a disease process in susceptible persons [1, 12]. There may be additional mutations in other genes that were not identified in our patient or as yet unidentified epigenetic factors that contributed to the aggressive nature of this child’s disease progression. 

## Conflict of interest 

The authors have no conflicts of interest to disclose 

**Table 1A.** ACTN4 gene mutations and variations [2, 15].

Disease causing or contributing	Nucleotide change	Amino acid change
Disease causing	175C>T	W59R
	del(445-447)	I149del
	763A>G	K255E
	776C>T	T259I
	784T>C* (This patient)	**S262P**
Probably not disease causing	929G>A	R310Q
	1046A>G	Q348R
	2401G>A	V801M
	2511G>A	R837Q
Non-disease causing	16C>G	A6T
	596A>G	None
	605T>C	None
	797T>C	None
	1292C>G	A427T
	1517T>C	None
	1920G>A	None
	2036C>T	None
	2400C>T	None
Unknown	1-34C>T	None
	184T>A	S62T
	657->C	Frameshift

**Table 1B.** Causes of collapsing glomerulopathy [13, 14].

Idiopathic		
Genetic	Non-syndromic	Familial
		Mitochondrial cytopathy (CoQ2 nephropathy)
		Sickle cell anemia
	Syndromic	Action myoclonus-renal failure syndrome
		Mandibuloacral dysplasia
Infections	Viral	Human Immunodeficiency Virus-1
		Parvovirus B19
		Cytomegalovirus
	Others	Campylobacter enteritis, Mycobacterium tuberculosis, Leishmaniasis
Autoimmune diseases		Systemic lupus erythematosus, Lupus-like syndrome
		Mixed connective tissue disorder
		Giant cell cerebral arteritis
		Still’s disease
Hematologic malignancies		Multiple myeloma
		Acute monoblastic leukemia
		Hemophagocytic syndrome
Drug exposure		Interferon-c
		Bisphosphonates
		Valproic acid
Post transplantation		De novo
		Recurrent
		Acute vascular rejection
Others		Thrombotic microangiopathy
		Severe hyaline arteriopathy (diabetic nephropathy, calcineurin inhibitor toxicity)
